# An Integrated Compensation Method for the Force Disturbance of a Six-Axis Force Sensor in Complex Manufacturing Scenarios

**DOI:** 10.3390/s21144706

**Published:** 2021-07-09

**Authors:** Lei Yao, Qingguang Gao, Dailin Zhang, Wanpeng Zhang, Youping Chen

**Affiliations:** School of Mechanical Science and Engineering, Huazhong University of Science and Technology, Wuhan 430074, China; rayyoh@hust.edu.cn (L.Y.); m202070756@hust.edu.cn (Q.G.); m202070578@hust.edu.cn (W.Z.); ypchen@hust.edu.cn (Y.C.)

**Keywords:** robot, six-axis force sensor, deep learning, least squares

## Abstract

As one of the key components for active compliance control and human–robot collaboration, a six-axis force sensor is often used for a robot to obtain contact forces. However, a significant problem is the distortion between the contact forces and the data conveyed by the six-axis force sensor because of its zero drift, system error, and gravity of robot end-effector. To eliminate the above disturbances, an integrated compensation method is proposed, which uses a deep learning network and the least squares method to realize the zero-point prediction and tool load identification, respectively. After that, the proposed method can automatically complete compensation for the six-axis force sensor in complex manufacturing scenarios. Additionally, the experimental results demonstrate that the proposed method can provide effective and robust compensation for force disturbance and achieve high measurement accuracy.

## 1. Introduction

Nowadays, robots have become indispensable equipment in industrial manufacturing systems [1]. With the urgent demand for flexible manufacturing and fast development of sensor technology, higher requirements are put forward for the intelligence and self-adaptability of robots [2]. Two prominent issues are robot-environment interaction [3] and human–robot collaboration [4,5]. The development of a multi-axis force sensor, especially a six-axis force sensor, makes it possible to obtain force information in robot operation environments. Additionally, six-axis force sensors have been applied to robotic systems that perform contact tasks, such as grinding robots, surgical robots, etc. [6,7].

In order to detect six-axis forces and moments in space, a cylindrical six-axis force sensor [8,9] is often used, which can measure three-axis orthogonal forces and moments relative to the sensor frame, i.e., the force components Fx,Fy,Fz along the X,Y,Z axes of the sensor and the moments Mx,My,Mz around the X,Y,Z axes of the sensor. When external forces are applied to the sensor, the internal elastomers are deformed and the corresponding strain signal according to its electrical characteristics are output [10,11,12]. Typically, a six-axis force sensor is mounted between the end flange of an industrial robot and the end-effector to perceive the external forces [13,14]. In this case, the sensor generally measures a combination of forces including the contact forces, the gravity force acting on the tool, self-gravity, system error, and other force disturbances. The tool gravity and self-gravity continuously affect measurement output as the robot pose changes. The self-gravity refers to the gravitational force acting on the six-axis force sensor. To accurately obtain the contact forces applied to the six-axis force sensor, it is necessary to compensate for force disturbance.

For several decades, researchers have been conducting wide studies to compensate for force disturbances of a six-axis force sensor at the end of a robot. Shetty B R et al. [15,16,17]. showed that the output of the sensor consisted of load gravity and interaction force, and derived an algorithm to estimate the effect of load gravity on the sensor output as the robot pose changed in real time. However, the algorithm was proposed under the assumption that the load gravity and the load centre of gravity are known, which is not always the case in practice. Loske et al. [18]. proposed an algorithm that considers the self-gravity of the sensor to be known and calculated the centre of self-gravity from the data of several poses in order to accomplish the compensation of the gravity. Lin et al. [19,20]. considered that the effect of gravity on the moment values can be ignored. Then, the output of the sensor in the six special attitudes were measured, and some parameters of the force sensor were initialized accordingly. Eventually, the gravity was decomposed when the sensor attitude changed, and thus the initial force and moment values were compensated. Li et al. [21]. completed the gravity compensation for the force interaction device using Lagrange’s equation. Vougioukas [22] assumed that the Z-axis of the robot base frame was in the same direction as gravity, and the gravity applied to one of the sensor’s axes was cancelled out by some special poses to calculate the bias of the force sensor. Finally, the load gravity and load centre of gravity positions were calculated by the force coordinate transformation and the least squares method. Taking into account the load gravity, the zero-point of the force sensor and the robot mounting inclination. Zhang et al. [23] proposed a method that used sensor data from no less than three robot poses to obtain the required parameters at once. However, the method assumed the zero-point as a constant and did not fully take into account the systematic error of the force sensor. Zhang et al. [24] used a deep learning algorithm to obtain the mapping relationship between the robot’s pose and the sensor output to complete the numerical compensation of a tandem force sensor. However, this method considers the tool gravity and sensor self-gravity as a whole, which requires data re-collection to train the model where the end-effector needs to be replaced. In addition, the method gradually fails when the end-effector wears out. This has been shown to be time-consuming and inefficient. Dine et al. [25]. presented a recurrent neural network observer to estimate the force disturbance due to gravity, inertia, centrifugal, and Coriolis forces. The method can detect external contact force-moment in a variety of highly dynamic motions. However, the result of the observer is unreliable when the robot is motionless, as the authors pointed out.

In order to solve the above problem, this paper proposes an integrated compensation method for the force disturbance of a six-axis force sensor, which combines deep learning and least squares. This algorithm separates the six-axis force sensor from the end-effector, and fully considers the self-gravity, drift, and system error of the six-axis force sensor. Firstly, the zero-point of the sensor is estimated based on deep learning to eliminate the interference caused by the above factors. Secondly, the load of the end-effector is identified using the least squares method. Finally, the influence on the output due to factors such as the gravity force acting on the tool and the robot mounting inclination can be eliminated based on model derivation, which is convenient for the cases where the end-effector needs to be replaced under complex manufacturing scenarios.

## 2. Problem Statement

A six-axis force sensor is often used for a robot to obtain contact force information. However, the self-gravity of the sensor and the gravity force acting on the end-effector will have an effect on the output of the sensor, and the influence changes continuously with the change of the sensor attitude. In addition, due to the inherent characteristics of electronic components, drift currents are changing, which affects the output of the sensor. Based on the above factors, the output of the six-axis force sensor differs from the pure contact force applied to the end-effector, and its output can be described as:

(1) $$f=f_{bias}+f_{o\_gravity}+f_{sys}+f_{t\_gravity}+f_{inertia}+f_{contact},$$

where $f_{bias}$ is the drift of the sensor, $f_{sys}$ is the system error, $f_{o\_gravity}$ is the effect caused by the self-gravity of the sensor, $f_{t\_gravity}$ is the effect caused by the gravity force acting on the tool, $f_{inertia}$ is the force output due to robot vibration and inertia, $f_{contact}$ is the pure contact force applied to the end-effector. In addition to the pure contact force, $f_{bias}$, $f_{sys}$, and $f_{o\_gravity}$ are parameters related to the sensor itself, $f_{t\_gravity}$ relies on the selected execution tool. Since the robot is in low speed, the effect of $f_{inertia}$ is not considered in this paper. Let the effects caused by the sensor itself be attributed to $f_{zero}$. Therefore, Equation (1) is rewritten as:

(2) $$f=f_{zero}+f_{t\_gravity}+f_{contact},$$

where $f_{zero}=f_{bias}+f_{o\_gravity}+f_{sys}$ is the output of the sensor without any external force, which is defined as the zero-point. The goal of the method is to calculate the zero-point of the sensor for different poses of the robot and the effect of the tool gravity on the output. Based on the above calculation results, the effect of force interference factors on the contact force perception is eliminated.

## 3. Integrated Compensation Method

In view of the effects of drift and system error, as well as the interference of the force sensor self-gravity and end-effector gravity, we need to compensate for the actual output of the force sensor in order to accurately perceive the contact forces. Therefore, we separate the force sensor and the end-effector, use deep learning to obtain the mapping relationship between the attitude and the zero-point of the force sensor, and use the least squares method to estimate the tool load. Then, the compensation of the force sensor is completed, and the influence of force interference is eliminated.

### 3.1. Zero-Point Estimation of a Six-Axis Force Sensor Based on Deep Learning

The defined zero-point includes the drift, system error, and the influence of its self-gravity. Generally, for simplicity, the drift is often regarded as a constant, and a linear function is established based on the relationship between the self-gravity and the posture. However, in real-life application scenarios of robots, the established linear relationship does not fully reflect the relationship between the posture and output as well as the effects of drift and system error due to the installation and positioning of force sensors, etc. Deep learning is a machine learning technique that has made significant progress in the past decade. With sufficient training data and suitable network structures, neural networks (*NNs*) can approximate arbitrary nonlinear functions, and this powerful fitting ability has been widely applied to natural language processing and image recognition [26,27,28,29]. Studies have shown that neural networks have a better effect on error compensation [30,31]. Given its capability described above, NN is used in this section to estimate the zero-point of the force sensor.

Thus, it is necessary to establish a mapping between the pose of the force sensor and the zero-point in the absence of any load on the force sensor, i.e.,

(3) $$f_{zero}=ℜ(p;\theta ),$$

where θ is the model parameter.

**Figure 1:**
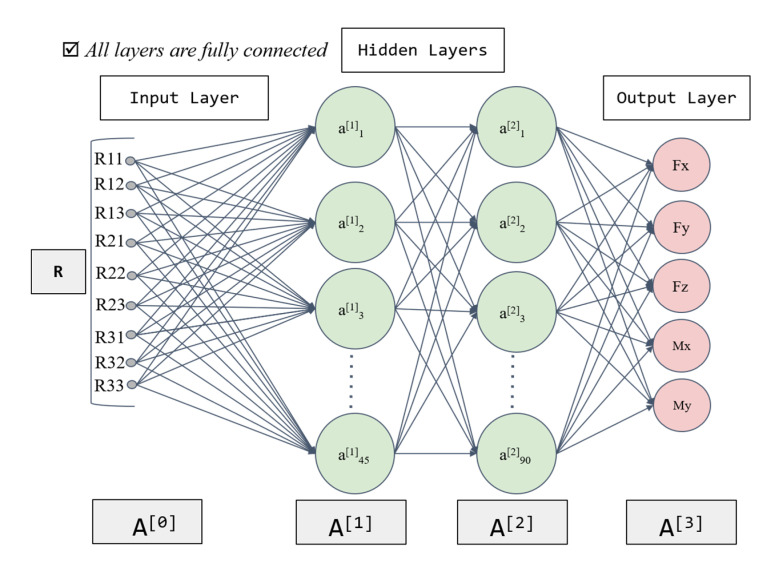
The neural network for zero-point estimation of a six-axis force sensor. A [0] is the input layer; A [1] is the first hidden layer; A [2] is the second hidden layer; A [3] is the output layer.

The pose of a rigid body can generally be described by rotation vector, rotation matrix, quaternion or Euler angles, etc. To reduce the computational complexity, the literature [15,16,22,23] chose a rotation matrix to derive the relationship between the effect of the self-gravity and the rotation matrix in the ideal case. According to the existing relationship, this paper designs an NN as shown in Figure 1 to estimate the zero-point.

The input to the NN is the nine elements of the rotation matrix $R_{ij}\;(i=1,2,3;j=1,2,3)$, and the general robot control system can directly obtain the data related to the pose and thus the rotation matrix can be calculated. Considering the symmetry of the force sensor, the theoretical output of Mz should be 0 in the non-load condition. Additionally, its output is small and fluctuates irregularly during the experiment. To reduce its unreliable effect on model training, the output data of the NN are the five outputs Fx,Fy,Fz,Mx,My of the force sensor, and the fluctuating output of Mz caused by incidental factors is not estimated. Theoretically, the accuracy of approximation can be improved by increasing the number of neurons by a moderate amount [32]. However, too many neurons require higher computation power, which inevitably affects the response time of the sensor in practice. Therefore, with a combination of fitting accuracy and sensor response speed, we chose a neural network with two hidden layers based on the experiments, the first with 45 nodes and the second with 90, as the regressor to fit the mapping function ℜ(p;θ). Currently, ReLU(x)=max(0,x) is often recommended as the activation function of NN, but in the force sensor application scenario, this activation function may cause some neurons to never be activated, resulting in the parameters not being updated. Therefore, this paper chooses the derivative of this function Leaky_ReLU(x)=max(αx,x) as the activation function, where α=0.01. The activation function of the output layer uses the constant function. The parameter θ in the model is learned by minimizing the following loss function:

(4) $$loss(\theta )=\frac{1}{N}\sum_{n=1}^{N}{\left(f_{zero}^{\left(n\right)}-{ℜ}^{\left(n\right)}(p;\theta )\right)}^{2}.$$

### 3.2. Tool Load Identification Based on Least Squares Method

Generally, different end-effectors are attached to the end of the force sensor in order to achieve various tasks. As the pose of the robot changes, the pose of the tool changes accordingly, and its influence on the sensor changes as well. In order to achieve a quick compensation of the gravitational influence of the end-effector after its replacement, this section uses the least squares method to estimate the tool load.

Let the robot base frame be {B} and the world frame be {W}, and assume that $Z_{w}$ is reversed with respect to gravity. Since the error in the robot installation will make {B} and {W} not completely coincide, assuming that {B} can be obtained by rotating {W} around the $X_{w}$ by angle α and around the $Y_{w}$ by angle β, then, the rotation matrix of the robot base frame relative to the world frame can be written as:

(5) RBW=[1000cosα−sinα0sinαcosα][cosβ0sinβ010−sinβ0cosβ].

**Figure 2:**
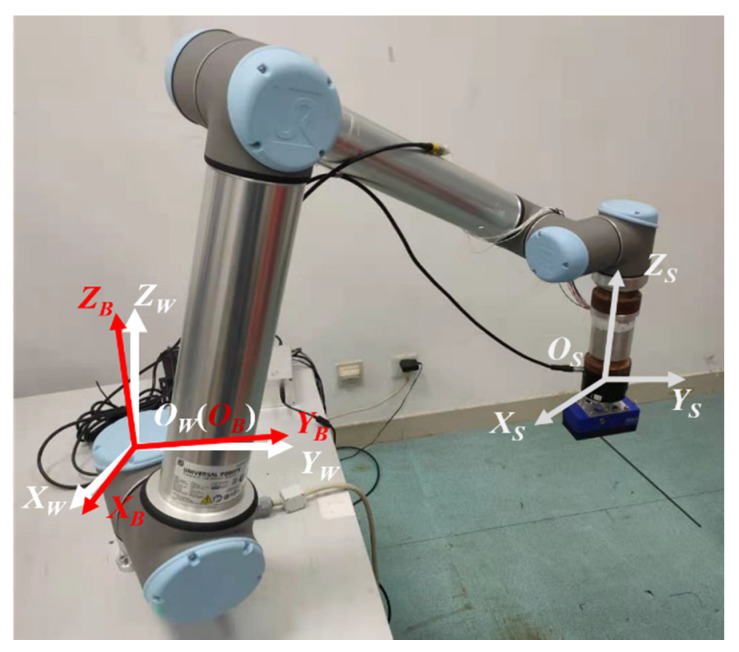
Relationship between the frames. {W} is the world frame; {B} is the robot base frame; {S} is the force sensor frame. *O* is the origin of the frame.

The frame of the force sensor is {S}, and the relationship between the frames is shown in Figure 2.

Assuming that the gravity force acting on the tool is *G*, then it is expressed in {W} as GW=[00−G]T. Through force transformation, the tool gravity in the *i*th attitude can be converted from the world frame to the force sensor frame:

(6) GiS=RiTSBRTBWGW,

which can be equated to:

(7) GiS=RiTSB⋅GB,

where GiS=[Gxi Gyi Gzi]T, GB=[Gcosαsinβ−Gsinα−Gcosαcosβ]T, and RSBi is the rotation matrix of the sensor frame at the *i*th pose with respect to the robot base frame.

**Figure 3:**
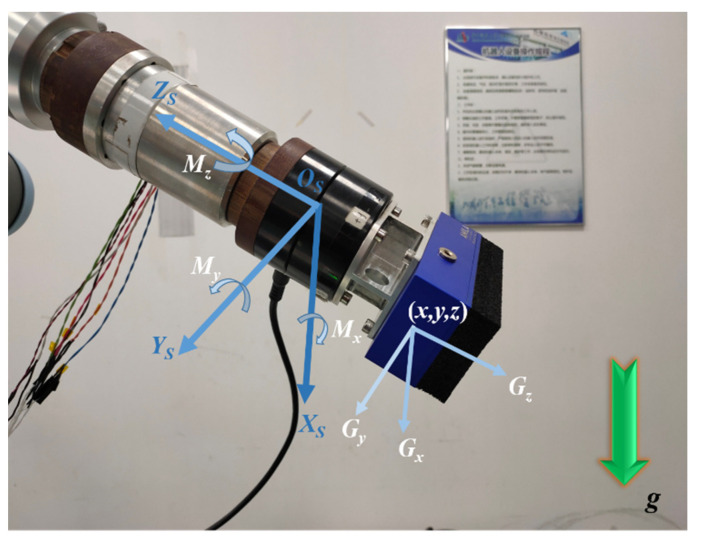
The gravity force acting on the tool in the force sensor frame. *g* indicates the direction of gravity; *Gx*, *Gy*, *Gz* are components of gravity; *Mx*, *My*, *Mz* are the component of the gravitational moment.

The gravity force acting on the tool in the force sensor frame is shown in Figure 3.

The frame of the six-axis force sensor is a spatial Cartesian rectangular frame. Assuming the coordinate of the tool’s centre of gravity in the sensor frame as (x,y,z), according to the relationship between forces and moments, it is obtained that:

(8) MSi=Air,

where MSi=[Mxi Myi Mzi]T, $r={\left[\begin{array}{ccc} x & y & z \end{array}\right]}^{T}$, and $A_{i}=\left[\begin{array}{ccc} 0 & G_{zi} & -G_{yi}\\ -G_{zi} & 0 & G_{xi}\\ G_{yi} & -G_{xi} & 0 \end{array}\right]$.

Stacking all forces GiS as GS, moments MSi as MS, rotation matrix RiTSB as R, and matrix $A_{i}$ as A in *n* robot poses, we have:

(9) GS3n×1=R3n×3⋅G3×1B,

(10) MS3n×1=A3n×3⋅r3×1.

Therefore, the gravity force acting on the tool and centre of gravity can be estimated as:

(11) G*B=argminGB(GS−R⋅GB)T(GS−R⋅GB),

(12) r*=argminr(MS−A*⋅r)T(MS−A*⋅r),

where $A^{*}$ is the matrix calculated from the optimal value of the tool gravity. Therefore, according to the least squares method, the optimal estimate of the load gravity and centre of gravity coordinates is:

(13) G*B=(RTR)−1RTGS,

(14) r*=(A*TA*)−1A*TMS.

### 3.3. Compensation of Force Disturbance

According to the proposed method, the zero-point $f_{zero}(p)=ℜ(p;\theta )$ of the force sensor is predicted by using the NN model when the robot is in an arbitrary attitude p. In addition, the optimal value of the tool gravity in the robot base frame G*B and the tool centre of gravity in the force sensor frame $r^{*}$ can be quickly estimated in a small number of poses. Further, when the robot is operated to an arbitrary attitude p, the effect of the tool gravity on the sensor output is calculated as follows:

(15) {GS(p)=RSBT(p)GB*MS(p)=A(p)r*ft_gravity(p)=[GS(p) MS(p)].

According to Equation (2), the pure contact force applied to the end-effector is:

(16) $$f_{contact}=f-f_{t\_gravity}(p)-f_{zero}(p).$$

Finally, the compensation of force disturbance can be completed according to Equation (16). See Appendix A for details of the pseudo-code of the proposed integrated compensation method.

## 4. Experimental Results

**Figure 4:**
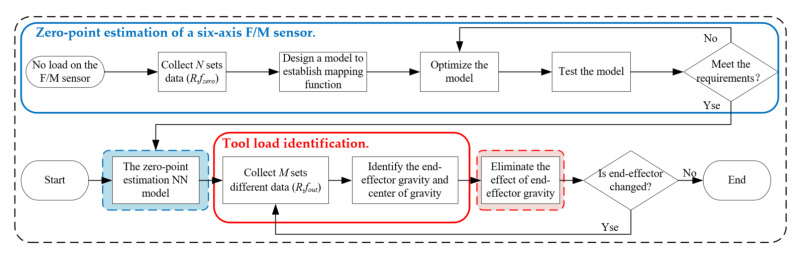
Flow chart of the experiment.

In order to verify the feasibility of the method, this paper designs the force interference compensation experiment of the six-axis force sensor at the end of the robot. The flow chart of the experiment is shown in Figure 4. The robot used in the experiment is the UR10 collaborative robot. The pose of the six-axis force sensor can be provided from the robot control system by positioning the installation of the six-axis force sensor and setting the position of the robot’s TCP (Tool Centre Point). The force sensor is a contact force sensor in a tandem force sensor [33] independently developed by our laboratory, using self-developed acquisition box and software for data collection. The technical parameters of the force sensor are shown in Table 1. A total of 200 sets of data are collected in each posture to ensure the stability and accuracy of the data, and eliminate the deviation caused by the robot vibration and inertia. Finally, the median value is taken as the response value in that posture.

### 4.1. Zero-Point Estimation of a Six-Axis Force Sensor Based on Deep Learning

**Figure 5:**
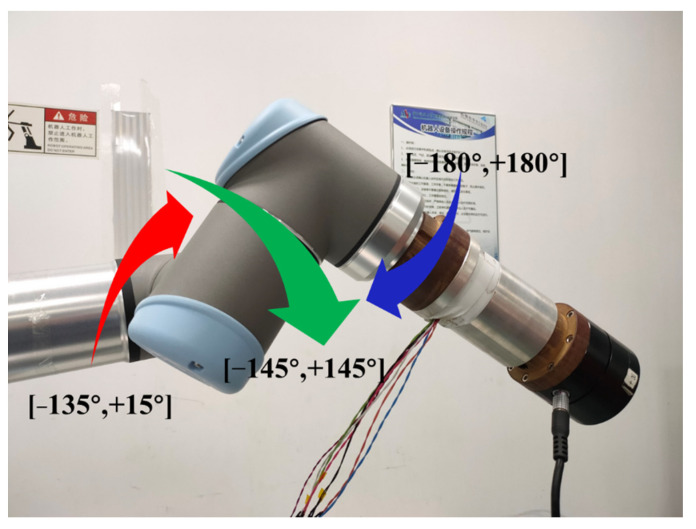
Illustration of the rotation of the joints. The red arrow represents the rotation of the fourth joint; the green arrow represents the rotation of the fifth joint; the blue arrow represents the rotation of the sixth joint.

It is necessary to collect sufficient output of the force sensor in different poses to learn the parameter θ, however, there is no smooth continuous trajectory that allows the robot to traverse all possible poses in the workspace. Xiong [34] et al. pointed out that the first three joints of the robot mainly determine the location of the wrist, and the latter three joints mainly determine the posture of the wrist. Considering the time required to collect the data so that the robot can traverse as many poses as possible while moving at a small angle each time, the following experimental scheme is designed. Keeping the first three joints of the robot fixed and considering the interference problem of the force sensor during the motion and the common working range, the data acquisition range of the fourth, fifth and sixth joints of the robot is restricted to $\left[-135^{\circ },+15^{\circ }\right]$, $\left[-145^{\circ },+145^{\circ }\right]$, $\left[-180^{\circ },+180^{\circ }\right]$ respectively, as shown in Figure 5. The step size of the three joints during data collection is $10^{\circ },10^{\circ },15^{\circ }$, so a total of 15∗29∗24=10,440 sets of data are collected. Each set of collected data includes the robot’s pose and the output of the force sensor in the current pose. After the original data collection, the order of the data is disordered randomly, and 9966 sets of data are selected as the training set, while the remaining 474 sets are used as the test set.

**Figure 6:**
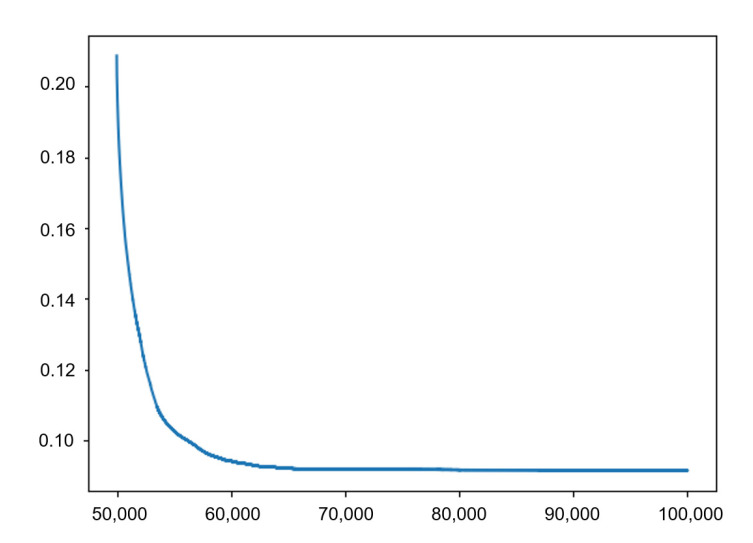
Loss function change curve of 50,000th–100,000th iteration.

The experimental environment is as follows: CPU is Intel(R) Core (TM) i7-9750H CPU@2.60 GHz, graphics card is GTX1660Ti, operating system is Windows 10, and Pytorch deep learning framework is used. The complete training data set of 9966 is used in each iteration, and the optimizer uses Adam with 100,000 iterations. The learning rate settings for the iterations are shown in Table 2. To prevent overfitting, L2 regularization is added to the loss function. Figure 6 shows the change of loss function for the 50,000th–100,000th iteration during the training.

**Figure 7:**
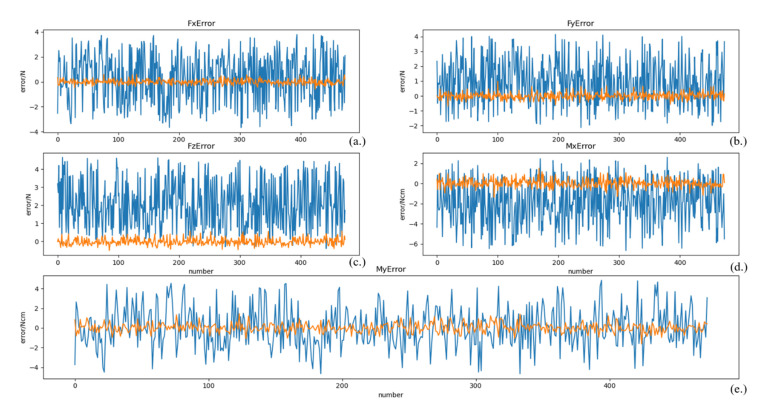
Zero-point estimation errors of the force sensor based on deep learning. (**a**) the estimation error of *Fx*, (**b**) the estimation error of *Fy*, (**c**) the estimation error of *Fz*, (**d**) the estimation error of *Mx*, (**e**) the estimation error of *My.* The blue lines represent the response values, and the orange lines represent the error.

**Figure 8:**
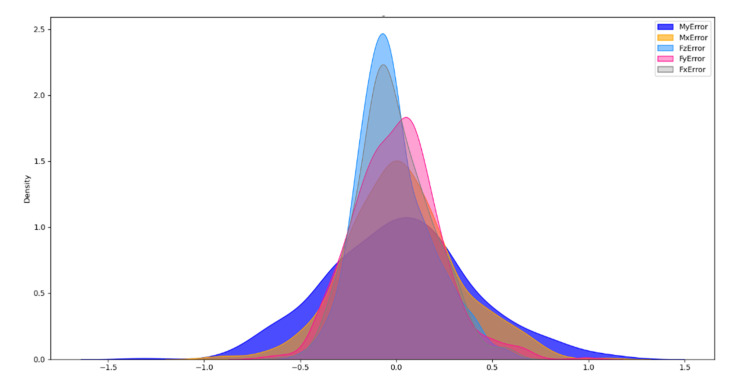
Kernel density plot of errors.

**Figure 9:**
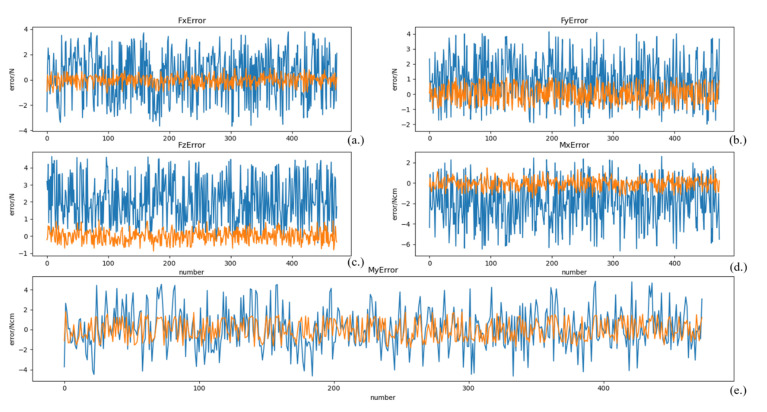
Zero-point estimation errors of the force sensor based on DM. (**a**) the estimation error of *Fx*, (**b**) the estimation error of *Fy*, (**c**) the estimation error of *Fz*, (**d**) the estimation error of *Mx*, (**e**) the estimation error of *My.* The blue lines represent the response values, and the orange lines represent the error.

After training, the model is tested using the test set data, and the results are shown in Figure 7, where the unit of forces is Newton (N) and the unit of moments is Newton-cm (Ncm). To illustrate the distribution of the zero-point estimation errors, a kernel density distribution of the errors was made, as shown in Figure 8. For comparison, a zero-point estimation method of the force sensor based on a dynamical model (*DM*) is conducted, and the results are shown in Figure 9. The mean ($\mu_{error}$), standard deviation ($\sigma_{error}$), and maximum value of the absolute value of the error ($\max_{error}$) obtained from the data are shown in Table 3.

Compared to Figure 9, the mean value of the error data on the test set in Figure 7 is close to 0. As shown in Table 3, $\max_{error}$ of the test dataset of the NN method is much less than that of the DM method, except for *Mx*, but the errors are extremely similar. In particular, the $\sigma_{error}$ of the NN method is much smaller than that of the DM method. The data shows that the error data distribution of the NN method is more concentrated and uniformly disordered on both sides of 0, as can also be illustrated by Figure 8.

**Figure 10:**
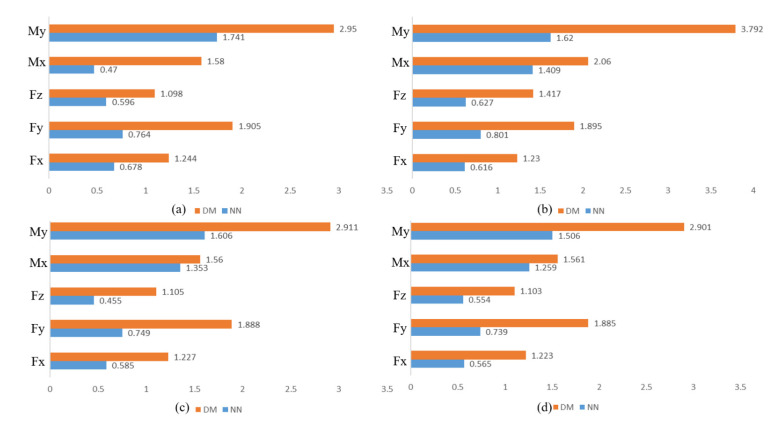
$\mu_{error}+3\sigma_{error}$ for different training data sizes. (**a**) The results of 1000 sets of training data. (**b**) The results of 3000 sets of training data. (**c**) The results of 5000 sets of training data. (**d**) The results of 7000 sets of training data.

To verify that the NN method outperforms the DM method, we reduce the size of the training set for experiments. The experimental results are presented in Appendix B. The bar graphs of $\mu_{error}+3\sigma_{error}$ are shown in Figure 10 for training data of 1000, 3000, 5000, and 7000 sets, respectively. Figure 10 shows that the DM method still performs much worse than the NN method even with the same training samples. Furthermore, the result shows that the NN model generalizes well and the errors obtained after fitting are caused by noise, so the deep learning-based sensor zero-point estimation method is feasible.

### 4.2. Tool Load Identification by Least Squares Method

**Figure 11:**
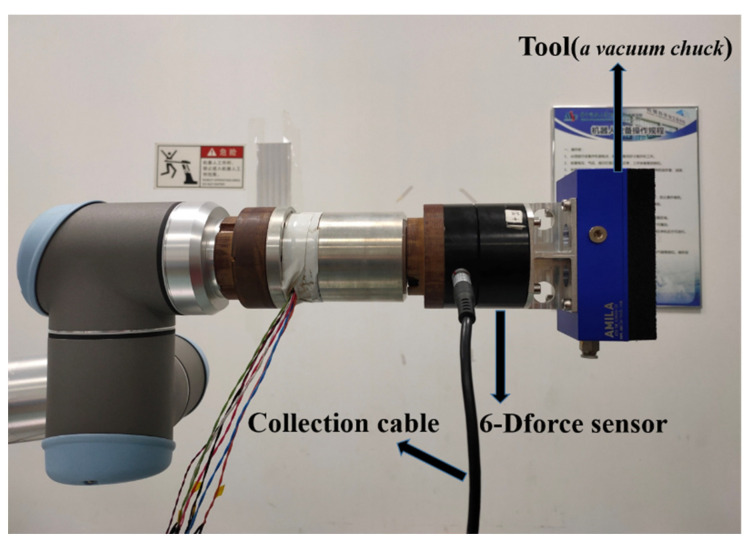
Data acquisition site with tool load for least squares method.

In order to verify the feasibility of the least squares-based tool load identification algorithm, a vacuum chuck is installed at the end of the six-axis force sensor to perform the experiment. The experimental site is shown in Figure 11. The robot is controlled by the host computer to adjust to 15 attitudes (rotation vectors) to collect data, respectively. The attitude data are shown in Appendix C.

The collected data are pre-processed and then calculated using the proposed method in Section 3.2 settlement results show G*B=[0.18 0.11 −5.11]T(N), $r^{*}={\left[0.037\;0.057\;-4.83\right]}^{T}(\mathrm{cm})$, calculated as expected. In reality, the mass of the vacuum chuck is 571 g, the gravity is 5.592 N. The gravity obtained from the identification is |G*B|=5.117 N, the error of the identification result is less than 8.5%. However, because the true value of the position of the centre of gravity cannot be measured accurately, and it is only used as the intermediate value in the compensation process, the error of the obtained result is not analysed. Therefore, we used Equations (15) and (16) in Section 3.3 to calculate the compensation results to analyse the feasibility of the method.

### 4.3. Compensation Results

When the tool at the end of the six-axis force sensor is not in contact with any object, the sensor does not receive any external force except for gravity, that is $f_{contact}=0$. According to Formula (16), the error after the compensation is:

(17) $$error=f-f_{zero}-f_{t\_gravity}.$$

**Figure 12:**
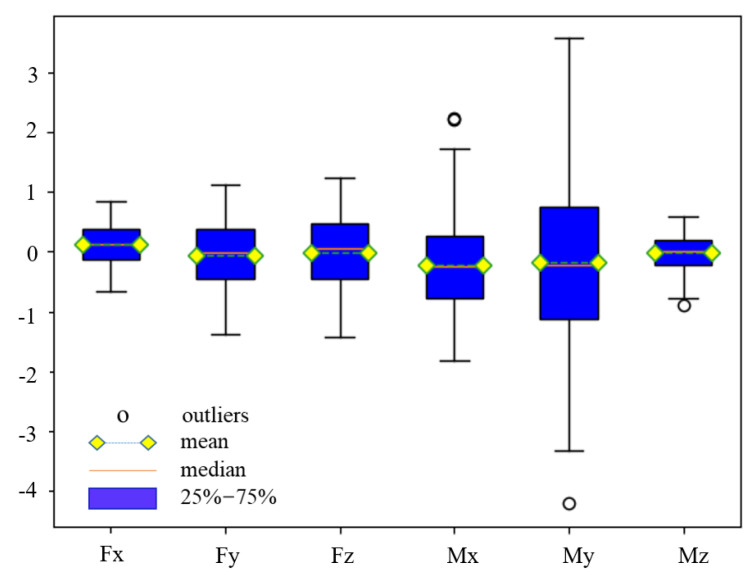
The box plot of test data.

According to the proposed method, the robot is adjusted to a series of random postures in the work area and collect force sensor data to verify the feasibility of the algorithm. The robot is controlled to move to 215 different random poses in the work area, and the corresponding robot pose data and force sensor output data are collected. The error between the response values f of the force sensor and the predicted values of the algorithm $f_{zero}+f_{t\_gravity}$, i.e., the compensation error of the disturbance force, is calculated and the box plot of the error is shown in Figure 12. The figure shows that the error distribution of the test data is relatively concentrated, and the percentage of abnormal data is less than 0.9%. However, the error values of *Mx* and *My* are large relative to the forces, which is due to the fact that they are influenced by both the forces and the load centre of gravity, resulting in an unavoidable accumulation of errors.

Table 4 shows the maximum absolute error (MAX) and the mean absolute error (MAE) of the compensation, where Bias+LSM is the compensation algorithm proposed in the literature [22], Double-LSM is the method proposed in the literature [23], which considers the sensor and the tool load as a whole, and NN + LSM is the proposed method. Table 4 shows that, compared with Bias + LSM and Double-LSM, the proposed method in this paper can effectively reduce the MAX and the MAE of Fx,Fy,Fz,Mx,My after the compensation. The compensation error level of Mz is comparable to that of the Double-LSM, which is due to the fact that the zero-point estimation in this algorithm does not compensate for Mz and only compensates for it in the load gravity identification stage. However, in general, the present algorithm can accomplish the compensation of robot end force disturbance with higher accuracy.

According to the technical parameters of the force sensor provided in Table 1, the errors after the compensation is completed by this method are 0.83%F.S., 1.39%F.S., 0.71%F.S., 0.45%F.S., 0.84%F.S., and 0.19%F.S., respectively.

To quantify the degree of error more accurately, the disturbance force compensation degree factor $\phi_{i}$ is defined as:

(18) $$\phi_{i}=\frac{\underset{j}{\max}(|n_{i}|)-MAX_{i}}{\underset{j}{\max}(|n_{i}|)}\times 100\%,\;i=1,2,\ldots ,6,\;j\in [1,length(test\;dataset)]$$

where $n_{i}$ is the output of the *i*th axis in the test. Therefore, this method reduces the effects of force sensor self-gravity, drift, system error, and tool gravity on the sensor output by 89.33%, 83.83%, 83.83%, 92.91%, 82.89%, and 76.48% in each dimension of the output, respectively. Through analysis, we conclude that the factors leading to incomplete compensation are: robot control error, the influence of the collection cable during the collection process, the calibration error of the six-axis force sensor, and the random error during collection. However, the compensated force error is still controlled within 1.50 N (1.5%F.S.) and the moment error is controlled within 4.3 Ncm (0.86%F.S.) by this algorithm. Compared to the literature [24], the present algorithm is able to perform the compensation task efficiently while meeting the accuracy requirements. Although the test was conducted for the particular robot and end-effector, the method is general.

In conclusion, compared to existing methods, the proposed method can significantly reduce the influence of the end of the robot interference force, and can meet the demand of active compliance control.

## 5. Conclusions

In this paper, we proposed an integrated method to compensate for the external forces applied to the six-axis force sensor. Considering the interference of drift, system error, and gravity, the proposed method used a deep learning model to predict the zero-point of the sensor and identified the tool load by using the least squares method. In the experiment, we designed and trained an NN model to predict the zero-point of the sensor, based on a 9966-item dataset obtained in the non-load condition. Moreover, we collected 15 groups of data when the robot arm was in different poses, including robot poses and sensor output. Such data were used to identify the tool load based on the least squares method. Finally, the force compensation values for the sensor were calculated by integrating the zero-point prediction and tool load identification obtained above. The experiment results show that the proposed method can perform more accurate and effective force compensation in different conditions compared with existing methods. This method can automatically complete force compensation of the six-axis force sensor in complex manufacturing scenarios, which can significantly improve the intelligence of the robot.

## Figures and Tables

**Table 1:** The technical parameters of the force sensor.

Type	*Fx* (N)	*Fy* (N)	*Fz* (N)	*Mx* (Ncm)	*My* (Ncm)	*Mz* (Ncm)
Range	±100	±100	±200	±500	±500	±500
Type I error	≤1%F.S.					
Type II error	≤2%F.S.					
Overload	120%F.S.					
Resolution	16-bit AD					
Temperature	−10~40 °C					
Humidity	20%~70% RH					

**Table 2:** Learning Rate.

Number of Iterations	Learning Rate
0th–10,000th	0.005
10,000th–30,000th	0.001
30,000th–50,000th	0.0005
50,000th–100,000th	0.0001

**Table 3:** Force sensor zero-point estimation error data.

Type	*NN*			*DM*		
	$\mu_{error}$	$\sigma_{error}$	$\max_{error}$	$\mu_{error}$	$\sigma_{error}$	$\max_{error}$
*Fx*	−0.009	0.193	0.607	−0.030	0.418	1.041
*Fy*	0.002	0.221	0.987	0.023	0.619	1.152
*Fz*	−0.009	0.165	0.549	0.003	0.368	0.958
*Mx*	0.037	0.405	1.040	−0.043	0.537	1.493
*My*	0.022	0.473	1.458	−0.054	0.984	1.823

**Table 4:** Comparison of compensation error results.

Types		*Fx* (N)	*Fy* (N)	*Fz* (N)	*Mx* (Ncm)	*My* (Ncm)	*Mz* (Ncm)
MAX	Bias + LSM [22]	1.368	1.826	1.735	3.340	5.721	1.036
	Double-LSM [23]	1.273	1.764	1.629	3.191	5.591	0.899
	NN + LSM	0.834	1.390	1.418	2.235	4.215	0.932
MAE	Bias + LSM [22]	0.502	0.583	0.724	1.211	2.356	0.430
	Double-LSM [23]	0.473	0.571	0.656	1.092	2.141	0.246
	NN + LSM	0.301	0.465	0.502	0.652	1.104	0.247

MAX, the maximum absolute error; MAE, the mean absolute error; LSM, least squares method; DNN + LSM, integrated method proposed in this paper.

## Data Availability

The data presented in this study are available on request from the corresponding author.

## Appendix A

The pseudo-code of the proposed method in this paper is shown in Algorithm A1.

**Algorithm A1:** Integrated compensation method.
**Input:**p: Current pose of the force sensor. {P}: Set of robot poses to be moved. ℜ(p;θ): Weight’s file generated by deep learning in Section 3.1. **Output:**$f_{contact}^{p}$: In the pose p, the pure contact force applied to the end-effector. **repeat** **if** end-effector is replaced **then** **for** *i* **to** *length* ({P}) **do** Control the robot move to the pose $p^{i}$ in {P}; Save current pose information RSBi in R; Save current output of the force sensor $f_{out}^{i}$ in $f_{out}$; Save current zero-point $f_{zero}^{i}=ℜ(p^{i};\theta )$ in $f_{zero}$; **end for** [GS MS]=fout−fzero; GB*=(RTR)−1RTGS; Calculate $A^{*}$ from GB*; r*=(A*TA*)−1A*TMS; **end if** **while** *p* is changed **do** Current output of the force sensor $f_{out}^{p}$ Get the rotation matrix at this pose RSBp; Zero-point of the force sensor at this pose $f_{zero}^{p}=ℜ(p;\theta )$; Effect of the end-effector: GSp=(RSBp)TGB* Calculate $A^{p}$ from GBp MSp=Apr* ft_gravityp=[GSp MSp] $f_{contact}^{p}=f_{out}^{p}-f_{t\_gravity}^{p}-f_{zero}^{p}$ **end while** **until** end of manufacturing task.

## Appendix B

Results for training data of different sizes. NN stands for Neural Network method and DM stands for Dynamic Model method.

**Table A1:** Error results obtained from training sets of different sizes.

Data Size	Type	*NN*				*DM*			
		$\mu_{error}$	$\sigma_{error}$	$\max_{error}$	$l_{error}$	$\mu_{error}$	$\sigma_{error}$	$\max_{error}$	$l_{error}$
1000	*Fx*	0.006	0.224	0.841	0.678	−0.010	0.418	1.027	1.244
	*Fy*	−0.001	0.255	0.808	0.764	0.051	0.618	1.158	1.905
	*Fz*	−0.019	0.205	0.531	0.596	−0.006	0.368	0.951	1.098
	*Mx*	0.038	0.144	1.493	0.470	−0.040	0.540	1.509	1.580
	*My*	0.028	0.571	1.783	1.741	0.001	0.983	1.841	2.950
3000	*Fx*	−0.008	0.208	0.682	0.616	−0.024	0.418	1.043	1.230
	*Fy*	−0.018	0.273	0.925	0.801	0.038	0.619	1.146	1.895
	*Fz*	−0.006	0.211	0.585	0.627	0.316	0.367	0.953	1.417
	*Mx*	0.062	0.449	1.526	1.409	0.452	0.536	1.488	2.060
	*My*	−0.003	0.541	1.493	1.620	0.837	0.985	1.812	3.792
5000	*Fx*	−0.006	0.197	0.602	0.585	−0.027	0.418	1.041	1.227
	*Fy*	−0.019	0.256	0.892	0.749	0.031	0.619	1.139	1.888
	*Fz*	−0.139	0.198	0.592	0.455	0.004	0.367	0.960	1.105
	*Mx*	0.048	0.435	1.331	1.353	−0.045	0.535	1.483	1.560
	*My*	−0.005	0.537	1.363	1.606	−0.041	0.984	1.810	2.911
7000	*Fx*	0.019	0.182	0.616	0.565	−0.031	0.418	1.045	1.223
	*Fy*	0.004	0.245	0.858	0.739	0.028	0.619	1.147	1.885
	*Fz*	−0.019	0.191	0.550	0.554	0.002	0.367	0.956	1.103
	*Mx*	0.020	0.413	1.159	1.259	−0.044	0.535	1.487	1.561
	*My*	0.045	0.487	1.675	1.506	−0.051	0.984	1.819	2.901

where $l_{error}=\mu_{error}+3\sigma_{error}$.

## Appendix C

The robot postures data required in the experiment of tool load identification based on least squares method.

**Table A2:** The robot postures data required in Section 4.2.

Number	*RX*	*RY*	*RZ*
1	2.0592	0.6381	−0.0558
2	0.6526	−2.7875	0.2766
3	1.8874	−2.0127	−0.2661
4	0.4880	2.1373	0.2597
5	0.4232	−1.8506	−0.6033
6	−1.9615	1.9499	0.2685
7	1.5291	−1.1463	−1.6160
8	−2.6872	−0.6012	−0.4095
9	−1.2868	1.6152	0.1995
10	−0.7769	2.4489	1.6684
11	−1.9852	0.5888	0.2570
12	−0.5084	−2.1222	−1.6644
13	−0.4566	−1.9525	−1.8284
14	−0.7513	−0.2524	0.6352
15	−0.8525	−2.4366	0.7001

*RX*, *RY*, and *RZ* are the three elements of the rotation vector.
